# Requirements for a maritime transition in line with the Paris Agreement

**DOI:** 10.1016/j.isci.2022.105630

**Published:** 2022-11-18

**Authors:** Sebastian Franz, Nicolas Campion, Sara Shapiro-Bengtsen, Rasmus Bramstoft, Dogan Keles, Marie Münster

**Affiliations:** 1 Technical University of Denmark, Department of Technology, Management and Economics, Energy Economics and Modelling, Kongens Lyngby, Denmark

**Keywords:** Energy management, Energy modeling, Energy policy, Energy resources, Energy sustainability

## Abstract

The shipping industry is a hard-to-abate sector in today’s society. Although past studies have looked at levels of carbon pricing, fuel savings, and the upscaling of green fuel availability separately, we combine these critical parameters for a green transition of the shipping industry to show what it takes to reach sectoral emissions reduction targets in line with the Paris Agreement. We utilize a least-cost optimization model drawing on data on, e.g., emissions with lifecycle elements and the costs of green fuel production. We find that reaching maritime reduction targets for a green transition requires high growth rates for green fuel availability, carbon pricing beyond 300EUR/tCO_2_eq, and at least 50% in fuel demand savings compared to today’s demand projection for 2050. The results show the importance of immediate climate action if maritime emissions reduction goals are to be achieved.

## Highlights

• Holistic least-cost optimization model of global long-distance shipping is developed
• Urgent transition of the maritime industry is required
• Carbon price of 300EUR/tCO2eq and demand savings beyond 50% of future demand required

## Graphical abstract

**Figure:**
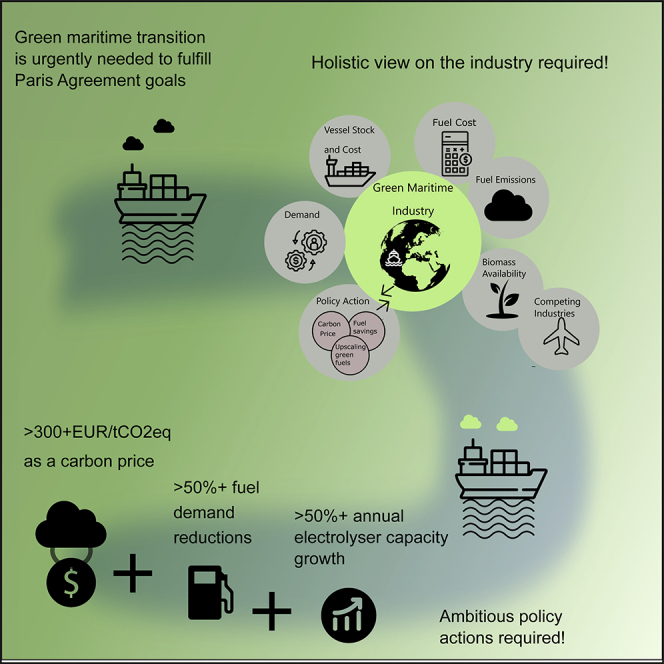

## Introduction

Reducing the emissions generated by the maritime sector is crucial to addressing the challenges of climate mitigation and meeting the Paris Agreement’s targets. The path we choose for the next century will decide whether we will overshoot the reduction goals to stay within a 1.5°C rise in global warming as set out by the Paris Agreement. It is essential to identify an efficient and feasible roadmap to avoid carbon-intensive lock-in for the maritime industry.^1^ Currently, the global maritime fleet is mainly fueled by Heavy fuel oil or very low sulfur fuel oil (VLSFO), maritime diesel oil (MDO), and partly by liquefied natural gas (LNG) (Smith et al., 2021). Switching to green fuels has been identified as having significant potential for GHG emissions reductions.^2^^,^^3^ The two primary green fuels, which are derived from green hydrogen and/or biomass, are methanol produced from carbon and green electrolytic hydrogen, and ammonia produced from nitrogen and green electrolytic hydrogen. The green transition towards a massive usage of green fuels depends, among other factors, on the scaling up of global electrolyzer capacity.^4^

There are several different detailed approaches to reducing emissions in the maritime industry, many of them involving carbon pricing and fuel savings as an essential policy and technological tool.^5^^,^^3^^,^^6^^,^^7^^,^^8^^,^^9^^,^^10^^,^^11^^,^^12^^,^^13^^,^^14^^,^^15^

Although past studies^5^^,^^6^^,^^14^^,^^15^^,^^16^ have looked at levels of carbon pricing, fuel savings, and the upscaling of green-fuel availability separately, we combine these critical parameters in a scenario for a green transition of the shipping industry to show what it will take to reach sectoral emission reduction targets in line with the Paris Agreement.

In this study, we push the research field by utilizing a least-cost optimization model, which encompasses (1) detailed fuel emission profiles featuring life-cycle elements, (2) costs of green fuel production, (3) constraints in upscaling green-fuel production capacities, (4) exogenous assessment of biomass availability, (5) emission reduction goals motivated by Paris Agreement narratives, and (6) two dimensions of climate action, namely carbon pricing and reduction in fuel demand. In doing so, we build on^2^^,^^3^^,^^4^^,^^17^ all of which highlight the challenging task of bringing the shipping industry onto a transition pathway in line with the pledges made under the Paris Agreement.

For this type of analysis, it is essential to define emission reduction targets that not only focus on achieving net-zero by 2050 (International Energy Agency, 2021b), but strictly use emission reduction targets to avoid overshooting (sectoral) carbon budgets motivated by the Paris Agreement.^18^ Thus, in this study, we used two maritime reduction targets to serve as a proxy for the 1.5°C global warming limited as formulated in the Paris Agreement. The two 1.5°C scenarios have been motivated by the IPCC narrative for a 1.5°C warming^19^ and actual emission pathways from the latest IPCC report.^20^ These reduction targets are more ambitious than that set out by the International Maritime Organization (IMO),^21^ yet they can be motivated by reduction targets as set out recently by leading shipping companies.^22^

This study can be broken down into several steps. Firstly, we model the optimal location for the production of green fuels to come up with bottom-up data on underlying emissions, costs, and resource usage (for more details, see fuels for the maritime industry and general assumptions for the e-fuel modeling process and Campion et al.^23^ After this, we investigate exogenous biomass availability scenarios for the maritime industry, which is essential in determining the availability of biofuels (for more details, see fuels for the maritime industry, exogenous biomass availability scenarios, and competing demand). In addition, we investigate the total cost of ownership and the availability of alternative engines for the shipping industry (for more details, see grey and blue ammonia and Sørensen et al.^24^). Finally, we combine these different modeling approaches and the resulting novel data to integrate them into one comprehensive least-cost optimization framework that allows us to analyze the dynamics and challenges of a green transition in the shipping industry.

We focus on the technological, economic, and environmental dynamics to transition towards a sustainable maritime industry. We use this holistic approach to distinguish the relationship between carbon-pricing efforts, savings in fuel demand, growth rates for green-fuel availability, and their transformational potential towards the Paris Agreement’s pledges. Results highlight the importance of early^25^ and efficient policy measures in international shipping to reach emission reduction goals in line with the Paris Agreement.

## Results and discussion

### Fossil fuels, biofuels, and e-fuels for the shipping industry

In this study, we model bottom-up Well-to-Wake (WTW) emissions, including upstream emissions and the underlying costs of, among other things, green ammonia (Haber–Bosch process using hydrogen from electrolysis), bio-e-methanol (biomass-to-methanol via thermochemical conversion boosted with electrolytic hydrogen), and e-methanol (CO2 hydrogenation using hydrogen from electrolysis and renewable CO2 from either direct-air-capture (DAC) or through point-source from a biomass-fired plant. (For more details, see Table S2 in the supplementary information). All green fuels are produced using 100% of solar PV and wind power without any connection to the public grid to ensure green electricity. The renewable power supply, fuel plant, and intermediate storage systems (hydrogen storage and batteries) are sized optimally to minimize the investment and operating costs for a given fuel demand. Four sites with good solar and wind profiles (Northern Chile, Western Sahara, northern Europe and Australia) are subject to testing, and the one with the lowest fuel production cost is chosen for reference. Compared to a system powered with grid electricity, the installed capacities need to be significantly oversized to satisfy the demand and avoid technical issues like frequent plant shut-downs. This is taken into account in the cost and WTW emissions analysis of green fuels (for more details, see Nami et al. 2021^26^ and Campion et al. 2021^27^).

**Figure 1:**
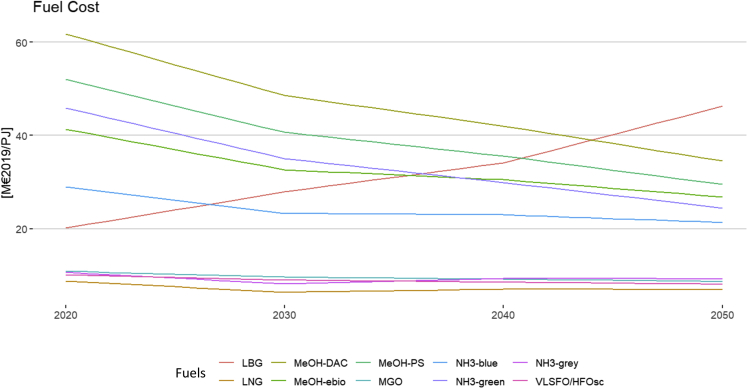
Blended fuel prices with electro-fuels powered using local renewable production

All analyzed green fuels take into account the pilot fuel oil, transportation to a central hub (Rotterdam) and profit margins (for more details, see general Assumptions for the e-fuel modeling process in the supplementary information). We show the final blended fuel cost in Figure 1. The respective fuels using biomass (LBG and MeOH-ebio) follow a sharp increase in the underlying biomass, as can be clearly seen for the LBG case. This price increase is motivated by global data from integrated assessment models (IAMs)^26^^,^^28^

The detailed bottom-up data related to green-fuel costs and WTW emissions is combined with a shipping stock model utilizing data on current and future engine technologies. We use data on the existing maritime fleet^29^ and additional options for investing in new ships (taking into account all costs related to the total-cost of ownership^24^), as well as options to invest in new engine types that could handle both green fuels and conventional fuels without retrofitting costs (see Grey and blue ammonia in supplementary information) Our bottom-up modeling approach allows us to implement learning curves and emission-intensity improvements to produce green fuels (see Figure 4). These improvements can be seen in the underlying cost and emissions data for the respective fuels (see General Assumptions for the e-fuel modeling process and Fuel prices).

### Ecological biomass potential and competing industries

One way to achieve the transition toward a sustainable maritime industry is to convert to biomass-based fuels. Some big players have already invested in a fueling pathway utilizing biogenic carbon to derive methanol (MeOH).^30^ Yet, the question of ecological biomass availability∗ remains, as this is linked to great uncertainty (30 EJ – 100 EJ). We use the midpoint in this range for our scenario analysis in this study. However, in the limitations section, we perform multiple sensitivity analyses for the upper and lower boundaries of the exogenous biomass availability.

**Figure 2:**
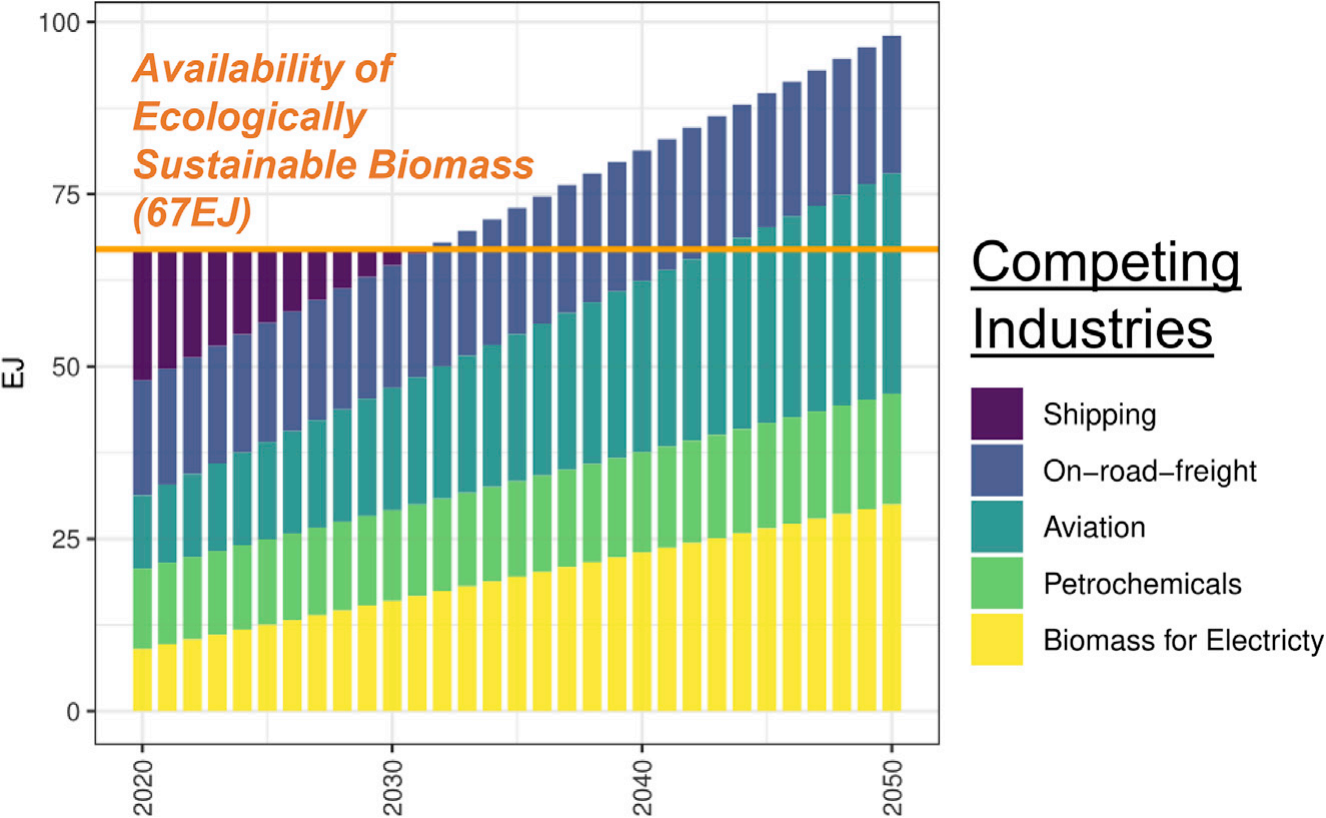
The availability and competition for ecologically sustainable biomass(*Ecologically sustainable biomass* = *the annual technical resource potential*, *taking into account the environmental sustainability aspects (for more details*, *see*supplemental information*section)*^†^ from different sectors increase the challenges of climate mitigation significantly by reducing the available ecological biomass for the maritime sector Availability data derived from (Gustafsson and Svensson, 2021; IEA Bioenergy, 2013; International Energy Agency, 2017, 2019; I.R.E.N.A., 2020; Oosterkamp, 2020); Data on competing industries from: Freight,^32^ Aviation,^33^ Petrochemicals,^34^ and Electricity.^35^ For more details, see Franz et al. 2021 (Franz et al., 2021).

Similarly, there is uncertainty regarding the competing utilization of biomass between different sectors. In this study, we include the sectors listed in Figure 2 (for more details, see Cost reduction of e-fuels). We use the midpoint scenario related to ecological biomass availability and competing demand (see Figure 2), but perform multiple sensitivities with other settings in Figure 6. We assume that other industries also utilize biomass, yet certain industries are not prioritized. Thus we show at what point the available biomass cannot fuel the demand and thus adjust the available biofuels accordingly. Our modeling approach is even more optimistic than other research suggesting there is no biomass in maritime applications.^31^ This analysis allows us to detail an available amount of biomass for MeOH-ebio, Liquefied Bio-Gas (LBG), or MeOH-Point Source (PS) fueling pathways.

### Understanding greenhouse gas emission accounting and carbon pricing

In our approach, we have two scopes for GHG emissions. One scope is related to the GHG emissions including life-cycle elements (only related to indirect emissions of fuel infrastructure) (GHG emissions including life-cycle elements = direct GHG emissions (Well-to-Wake) plus upstream emissions related to fuel infrastructure) of a particular fuel. In this scope, we count the emissions related to building the infrastructure (e.g., steel processing, concrete, mineral extractions) used to build wind towers, solar PV panels, batteries, electrolyzers and fuel plant emissions, and ending with the final fuel emissions during combustion. More details about all the underlying assumptions for the cost and emission derivation of the analysis green fuels can be found in the Supplementary Information (section General Assumptions for the e-fuel modeling process to Exogenous Biomass availability scenarios). As can be seen, the underlying assumption is that the entire production of green fuels will become carbon-neutral in the years to come. This assumption is motivated by several plans to reach net-zero by 2050 for producing sectors. This plays an essential role when looking at upstream emissions, as these are the hardest emissions to reduce.^36^^,^^37^

We do not take land-use changes into account, but these could be assessed in a further analysis.

Furthermore, we assume that the biomass used is CO2-neutral, as only residual biomass is included (for more details, see Figure 2, also Franz et al. 2021^38^).

**Figure 3:**
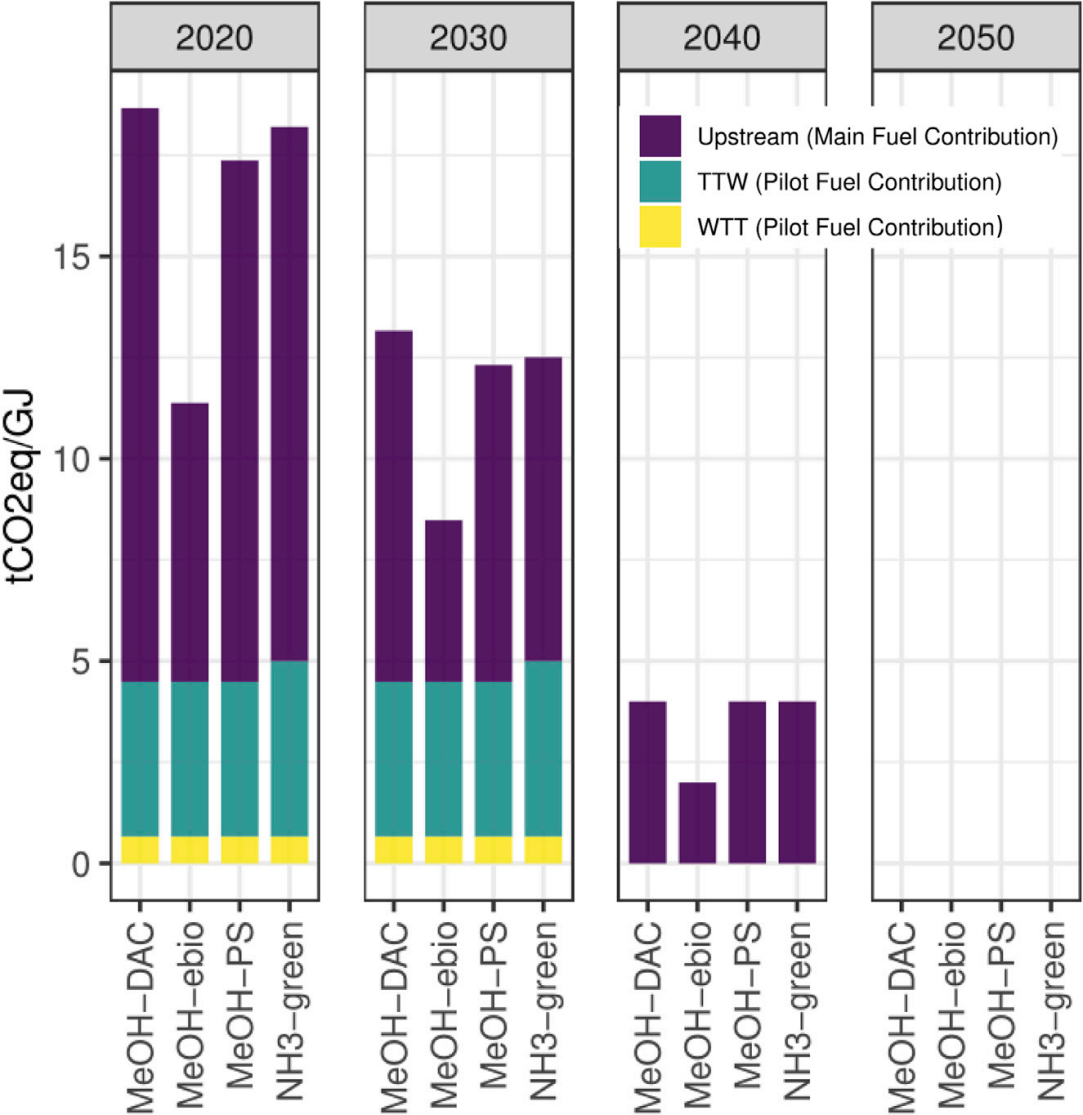
Emissions accounting for modeled green fuels blended with 5% pilot fuel (VLSFO in 2020/2030, DME in 2040/2050) TTW: Tank to Wake (Pilot Fuel Contribution, (NH3 also includes boil-off-gases)); WTT: Well to Tank (Pilot Fuel Contribution). Upstream: emissions related to infrastructure (power supply, fuel plant, and storage systems). For more details, see Nami et al. 2021^26^ and Franz et al. 2021.^38^

The second scope of GHG emissions-accounting in our approach is related to the taxed GHG emissions. We only tax the WTW emissions without the upstream emissions (see Figure 3; purple parts of bars have not been taxed in this study) for building the fuel plant and the power supply infrastructure to avoid biased results by applying a carbon price twice. This is because GHG emissions related to, e.g., steel production are one of national GHG reduction targets; it is assumed that these emissions have already been taxed at the local level (e.g., via the EU ETS scheme) or as a share of import tariffs, as discussed by the EU Commission^39^ they should therefore be excluded from the carbon taxation of the fuels. In both scopes, we assume no connection to the electricity grid. Thus, all the electricity used to derive green fuels comes directly from the respective fuel plant’s specific mode of renewable electricity generation (PV or wind, depending on the modeled location. For more detail, see^26^^,^^27^^,^^38^). This approach guarantees that green fuels are produced from physically traceable green electricity.

**Figure 4:**
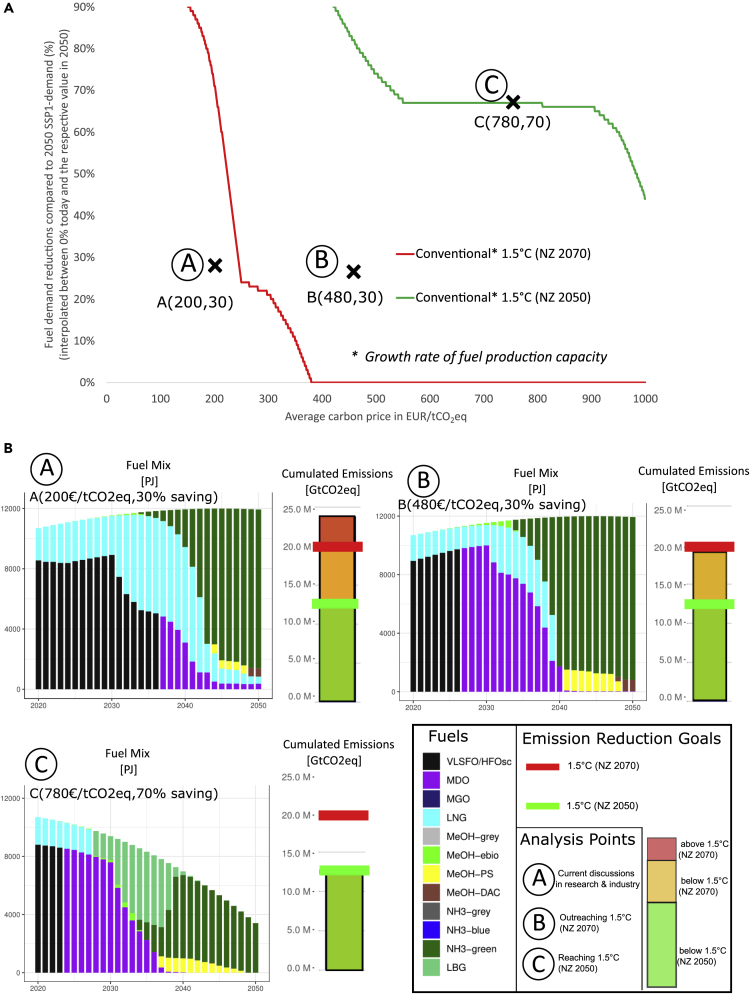
Global maritime mitigation pathways for certain levels of carbon pricing and fuel demand reductions (A) solution space to reach Paris Agreement pledges. (B) global maritime fueling pathways and cumulated emissions for certain combinations of carbon pricing and fuel savings.

Our bottom-up modeling approach allows us to implement learning curves and emission-intensity improvements to produce green fuels (see Figure 4). These improvements can be seen in the underlying cost and emissions data for each fuel (see General Assumptions for the e-fuel modeling process and Fuel prices). With this in mind, the way a carbon price is implemented is highly relevant, because we assume decreasing costs and emissions for green fuels (leading to zero emissions for Green Fuels in 2050 (see Figure 3)). Thus, the dynamics between revenues from carbon pricing and green transition efforts can be described as non-linear.

### Scenario design in a least-cost optimization framework

**Figure 5:**
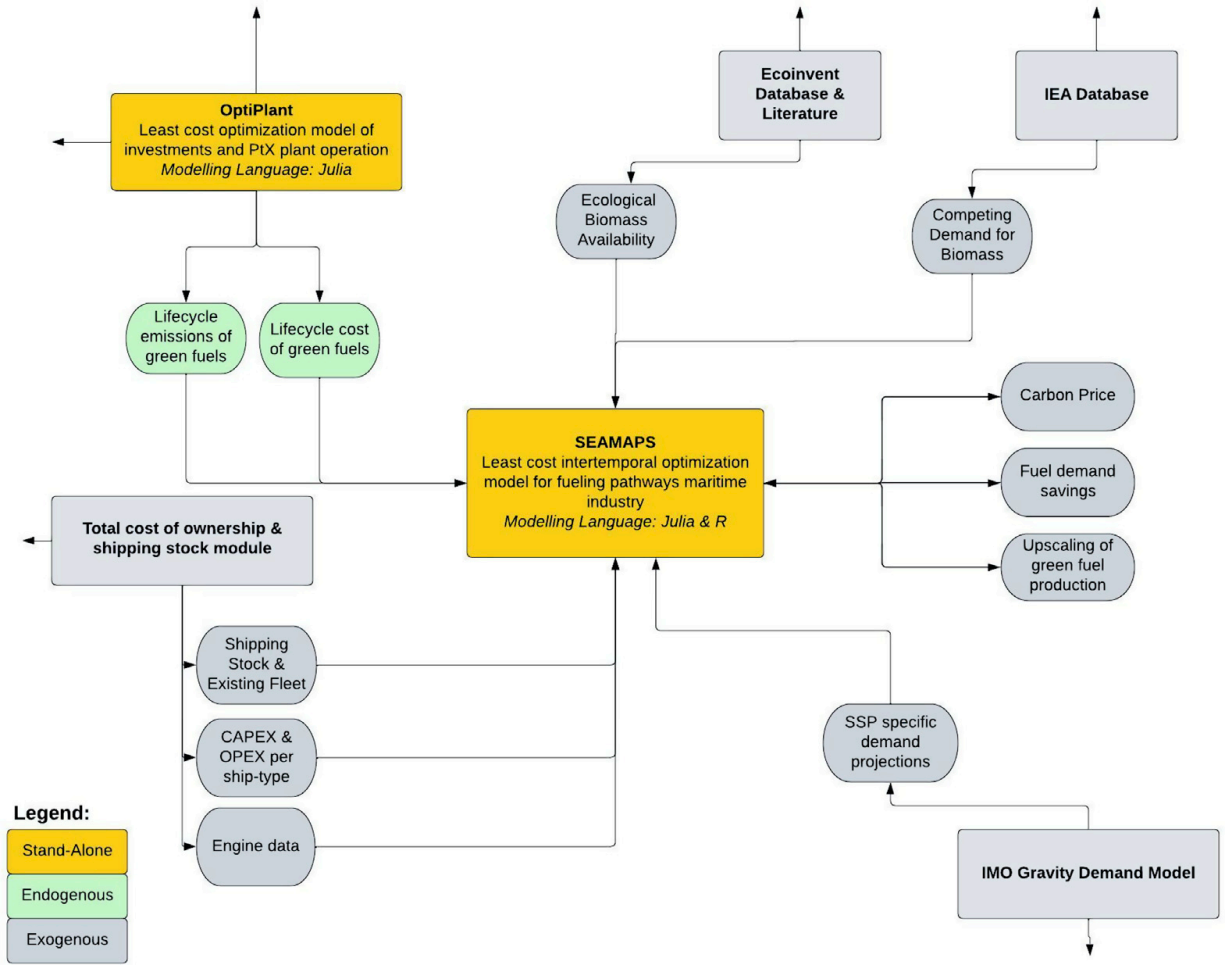
SEAMAPS modeling environment.

In this work, the SEAMAPS model^38^ (for more details, see STAR Methods section) is combined with two carbon-pricing schemes (see Table 1) to identify different green transition pathways (Figure 5).

**Table 1:** Scenario design of baseline scenario

Scenario Settings	Description
Carbon Pricing Scheme	Progressing Carbon Price ranging from 50EUR/tCO_2_eq in 2020 to varying carbon pricing (xx-nn EUR/tCO_2_eq) in 2050)
Scaling up Green Fuels	Conventional green fuel growth rate (50%/year) starting from 5PJ in 2020 and leading to exponential growth.^40^^,^^41^
Biomass Availability	Biomass Availability Constraint (x EJ in 2020 to n EJ in 2050) (see Figure 2)^38^
Fuel Demand	Shared Socioeconomic Pathway One (SSP1) - (IMO)^29^

In Table 1, we show the two baseline scenarios. For the reference scenarios, which should serve as a proxy for a 1.5°C global warming emissions reduction pathway, we used two different interpretations of the 1.5°C global warming emissions reduction goal (Table 2). The scenario called “1.5°C (Net Zero (NZ) 2050)” is motivated by the IPCC narrative for a 1.5°C global energy system mitigation pathway. The scenario called “1.5°C (NZ 2070)” is motivated by the maritime mitigation pathway for a 1.5°C compatible world in the latest IPCC report.^20^ The difference between the two scenarios thus lies in the level of detail for the sectoral mitigation pathway. Although “1.5°C (NZ 2050)” assumes the same mitigation profile for all sectors, regardless of the sector-specific mitigation challenges – which are significantly higher for maritime than, for example, for light-duty vehicles^4^– the “1.5°C (NZ 2070)” scenario shows the shipping sector’s emissions pathway set out by the IPCC to be in line with the Paris Agreement’s goal of 1.5°C global warming.

**Table 2:** Reference global warming scenarios in line with the Paris Agreement

Reference Scenarios	1.5°C (NZ 2050)	1.5°C (NZ 2070)
Short-Term GHG Emission Reductions	45% by 2030^19^	25% by 2030^20^
Reaching Net-Zero Emissions	100% by 2050^19^	100% by 2070^20^

### Reaching Paris Agreement emissions reduction goals

When analyzing how far future fueling pathways and cumulated emissions are in line with the Paris Agreement, we find contrasting transition pathways and associated challenges toward climate mitigation. For Figure 4, we ran the SEAMAPS model several hundred times with different values for carbon-pricing and reductions in fuel demand. These fuel-demand reduction measures could include a change in the contractual design explicitly banning the logistic practice “steam fast, then wait,” which incentivizes shipping companies to burn more fuel than necessary and thus emit up to 15% more globally scale.^42^ Another significant fuel-saving potential lies in the fuel transition toward locally produced green fuels thus decreasing fuel trading volumes now accounting for 45% of global shipped trade by weight.^43^

Furthermore, measures could be related to improvements in engine design, ship design, hydrodynamics, and slow steaming in general.^3^^,^^13^^,^^44^^,^^45^^,^^46^^,^^47^ Although carbon-pricing and reductions in fuel demand are only two possible measures, the magnitude of the challenge we are facing is evident. The ability to alleviate this involves reducing GHG emissions, including upstream emissions, and reducing the costs of green fuels, e.g., by accelerating the scaling-up of fuel production and the learning curves, thereby pushing down costs earlier. The reductions in fuel demand are implemented as a percentage reduction compared to SSP1 demand^29^^,^^48^^,^^49^ in 2050 and are linearly interpolated. The reduction in fuel demand is spread over a time period of 30 years (90% reduction compared to 2050 = 3% reduction per year compared to the SSP1 projection). Figure 4A shows the underlying interrelations between average carbon prices (x-axis) and fuel demand reductions (y-axis) for reaching either a 1.5°C (NZ 2050) or a 1.5°C (NZ 2070) emissions reduction target.

We find that achieving the maritime emissions reduction goals of limiting warming to 1.5°C (regardless of when we are predicted to reach net-zero) with a conventional growth in green-fuel production capacity of 50%^40^ requires more ambitious policy action than the 200EUR/ tCO2eq currently discussed in industry and research.^5^^,^^6^^,^^14^^,^^16^ In fact, in Figure 4A) we find that it either requires carbon-pricing beyond 300EUR/tCO2eq (on average within a 30-year horizon) and fuel savings of at least 30% in 2050 compared to SSP1 demand projections in 2050 , or carbon-pricing beyond 200EUR/tCO2eq but very ambitious fuel savings of up to 90% of total fuel demand in 2050.

To put the conventional growth (50% annual growth of electrolyzer capacity) of green-fuel capacity into context: historical solar PV capacity growth from 2009 to 2019 was between 24 and 89% per year, depending on the geographical region (low for EU, high for non-OECD); and historical wind-capacity growth from 2009–2019 was between 11 and 28% per year depending on the geographical region (low for EU, high for non-OECD) (BP, 2020). (For more details, see section 13 of the supplemental information, where we discuss different upscaling rates for capacities^50^) However, suppose it becomes more and more apparent that electrolytic green fuels are the only solution to achieving a green transition in hard-to-abate sectors. In that case, growth rates beyond solar PV growth, which at the time (2009–2019) was not and still is not the single option to invest in to generate renewable electricity, might be expected to meet or even to exceed the 1.5°C (NZ 2050) emissions reduction target.^50^

Assuming an unconventional growth rate (126% annual growth), which comes close to the diffusion speed of US nuclear weapons or World War II US aircraft,^40^ a 1.5°C (NZ 2050) emissions reduction goal is achievable with average carbon-pricing levels beyond 350EUR/tCO2eq and no fuel savings or a combination of fuel savings and average carbon pricing of 300EUR/tCO2eq. A 1.5°C (NZ 2070) emissions reduction goal can be achieved with significantly less climate action, namely, average carbon pricing of 250EUR/tCO2eq alone, or average carbon pricing of 150EUR/tCO2eq and 80% fuel savings in 2050. (These findings can be seen in Exogenous Biomass availability scenarios in the Supplemental information.)

To model exponential growth in the availability of green fuels, we assume it will increase from today’s value of around 5PJ^26^^,^^40^^,^^51^ per year at a growth rate of 50% (conventional) or 126% (unconventional). We thus assume that green-fuel capacity starts growing once the fuel is being invested in. Using both conventional and unconventional growth rates shows the impact of increasing the speed of green-fuel production, yet this should not be interpreted as a maximum or minimum; rather, it should serve as a guide within a sustainable narrative (SSP1-type narrative).

Figure 4B shows three fuel mixes that are related to the marked points in Figure 4A. Point A marks the current discussion around fuel-demand savings and carbon pricing.^5^^,^^6^^,^^14^^,^^15^^,^^16^ Point B exemplifies a fuel mix that is in line with the 1.5°C (NZ 2070) emissions reduction pathway, whereas point C shows a fuel mix that is just in line with the 1.5°C (NZ 2050) emissions reduction pathway. The future maritime fuel mix is based on the underlying carbon-pricing strategy. In the analyzed scenarios, we see many fossil fuels like VLSFO/HFOsc, especially LNG and MDO in the short to medium term. We identify green ammonia (NH3-green) as a predominant fueling option in the longer term. Above a carbon price of 300 EUR/tCO2eq, green ammonia becomes the cost-competitive fueling option and is thus utilized to a large extent.

Challenges and uncertainties regarding the operational feasibility of the fueling pathways described here exist in all scenarios. Yet, some fuels are more controversial than others. For example, blue ammonia (NH3-blue) could be used as a bridging fuel if it is considered to have a beneficial effect on global emissions compared to LNG^52^^,^^53^ and is made available on a large scale. Green ammonia could potentially fuel significant parts of the future global fleet if the concerns raised regarding the safety of ammonia as a marine fuel are solved.^6^^,^^54^ Ammonia is poisonous, explosive, and a potent fertilizer. Therefore, avoiding leakage into the air or the marine environment and ensuring its safe storage in harbors is essential. Furthermore, the indirect climate effects of hydrogen are starting to be discussed, not having been taken into account previously.^55^ If they were, the arrow might point to fewer electro fuels and higher fuel demand savings. Similarly, discussions are ongoing concerning whether residual biomass can be considered CO2-neutral, which would push toward more electrofuels. However, if safety concerns can be solved in the years to come, green ammonia may become the dominant form of renewable energy and ultimately the dominant marine fuel.^56^

These levels of carbon pricing and fuel-reduction potentials diverge significantly from the findings of current research and industry reports.^5^^,^^6^^,^^14^^,^^15^^,^^16^ This is because of our novel perspective on green fuels (including upstream emissions) and the optimization model's constraints on scaling up green-fuel production capacities. We performed a sensitivity analysis without these features and found that it is possible to reach net-zero by 2050 with a carbon price of less than 200EUR/tCO2eq and no fuel savings. However, the cumulated emissions will overshoot the defined 1.5°C emission reduction targets. This shows the weakness in using net-zero by 2050^35^ as an emissions reduction goal since cumulated emissions, and thus a sectoral carbon budget, are more critical than evaluating the emissions at one specific point in time to avoid overshooting the Paris Agreement goals.^18^ (true to the maxim, “sometimes it is more about the journey than the destination”).

### Limitations of the study

**Figure 6:**
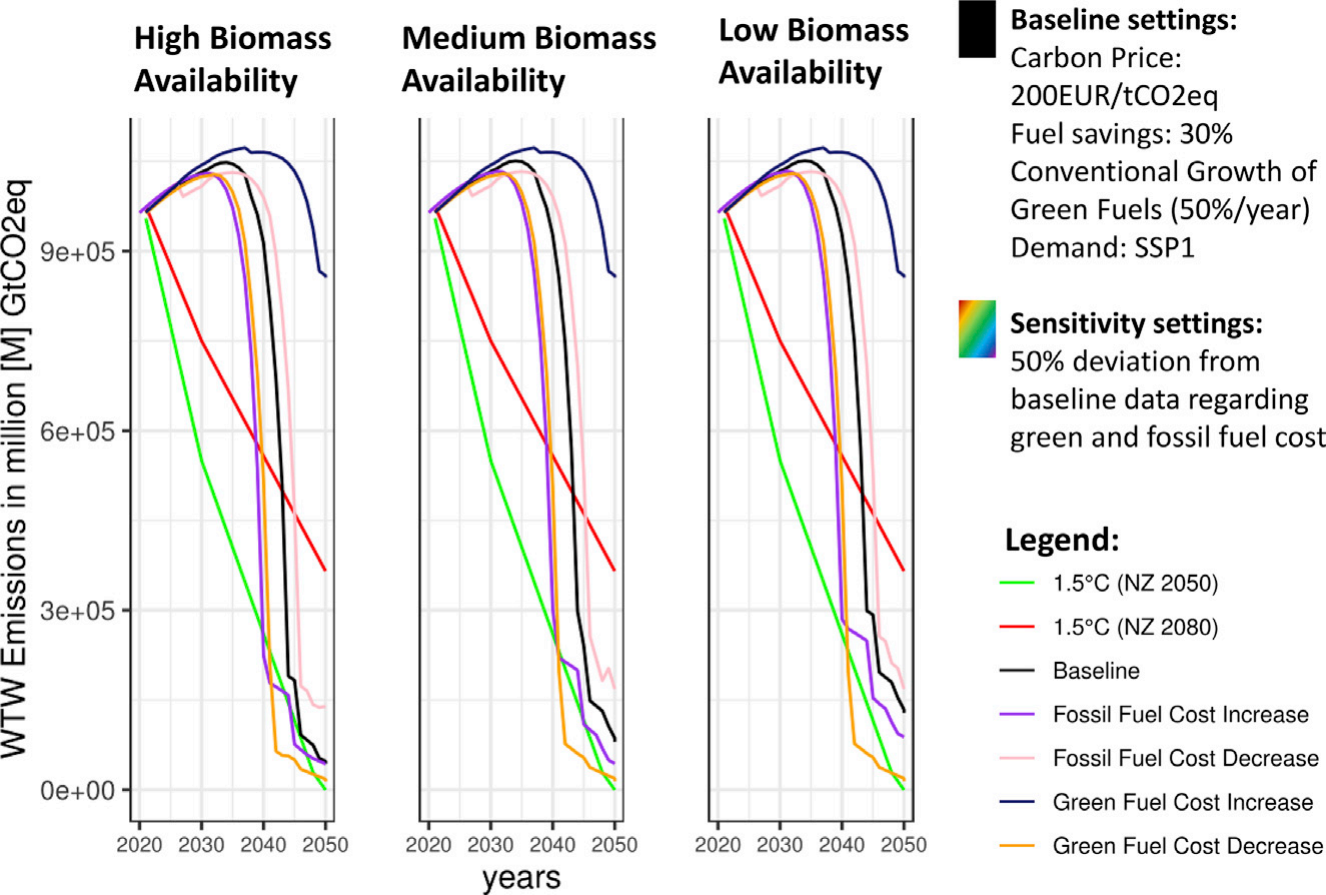
Sensitivity Analysis for main assumptions of the modeling approach

When modeling a global fleet of more than 60,000 vessels, including future fueling pathways, the development of certain technologies (e.g., CCS, DAC) and the availability of resources for the future, certain assumptions have to be made. In our case, the main assumptions are related to the cost development of green fuels and fossil fuels, the availability of biomass, and the growth of upscaling for specific technologies. In Figure 6, we show different sensitivity parameters that address the uncertainties with regard to the respective assumptions.

Figure 6 gives yearly emission profiles for different exogenous biomass availability scenarios and the main assumptions regarding fuel costs respectively. The underlying baseline scenario has been motivated by the current carbon pricing discussion (200EUR/tCO2eq)^5^^,^^6^^,^^15^^,^^16^ and feasibility studies for fuel savings (30% by 2050).^3^

One can see that different biomass availability scenarios do not necessarily change the emissions pathways significantly. The challenges of climate mitigation for the sector are slightly lower for the high biomass availability scenario because some biofuels like liquefied biogas (LBG) can serve as a bridging fuel in the early years of the modeled time horizon. However, this assumption has no significant impact, as biofuels are expected to increase in price significantly^28^^,^^31^ in the future because of increased competition from other sectors, making them less likely to be used by the maritime sector.

Unlike biomass availability, the different pathways and learning rates included in the assumptions and modeling of green fuels and fossil fuels do have a significant impact on future mitigation challenges for the maritime industry. In Figure 6, it is evident that rises in fossil-fuel prices of up to 50% compared to the baseline prices in Figure 6 lead to lower emissions, as green fuels will become competitive earlier given the underlying carbon price. Following this argument, this effect could be expected to occur in the real world, given the rise in fossil-fuel costs in the present and the near past.^57^ However, this effect also leads to a similar mechanism related to increased estimates of green fuel costs. In this case, the maritime industry's challenges regarding climate mitigation increase significantly.

Further research should focus on increasing the heterogeneity in different fueling pathways, both upscaling and emissions/cost-related. This feature could increase the level of detail significantly to allow us to draw a more precise picture of future fueling options. Furthermore, the perfect foresight constraint could be relaxed toward limited foresight by introducing rolling horizon investments. This additional feature would allow us to model the lock-in effects of specific fueling pathways, as we are now seeing long-term contracts in the maritime green-fuel supply.^58^ Another interesting topic to look into would be detailed modeling of future fuel demand savings.^3^ have already provided us with a sophisticated picture of possible fuel demand savings, suggesting that building on this work would be an interesting option. In addition to this, comparing the costs with the alternative use of fossil fuels offset by carbon capture storage (CCS) would be another exciting option to look at in further research.

### Conclusion

This analysis has shown (1) that the Paris Agreement will not be met without significant improvements in fuel demand savings, and (2) that very high CO2 prices are required if this is the only measure implemented for achieving the Paris Agreement. It is also argued that policy options designed to ramp-up key technologies, such as electrolysis, thereby increasing the upscaling to unconventional growth levels, would assist the transition tremendously. If global CO2 prices cannot be implemented, standards or fueling mandates and other sticks could replace them. However, the difference in price levels between green and fossil fuels is still very high that efforts such as carbon pricing alone seem unrealistic. However, with the rising rivalry in the world and thus rinsing prices for fossil fuels one could expect lower challenges toward mitigating this sector.

With this in mind, one can see that the shipping sector is at a crossroads, and the coming years will be decisive for future challenges regarding climate mitigation. We find that achieving emission reduction goals in line with a 1.5°C (net-zero 2070) warming scenario given a conventional growth rate (50% per year) for the upscaling of green-fuel production capacities requires a progressive carbon price of 50EUR/tCO2eq in 2020 increasing to 550EUR/tCO2eq in 2050 and fuel savings of at least 30% in 2050 compared to an SSP1 demand projection. The maritime emissions reduction target for a 1.5°C (net-zero 2050) warming scenario requires even more ambitious carbon-pricing and fuel-savings measures. However, in line with,^59^ we find that the future growth rate for scaling up green-fuel production capacities and the cost of green fuels are the most sensitive parameters for the future climate mitigation challenges of the shipping sector. They thus present a unique opportunity to bring the shipping industry onto a pathway that observes the 1.5°C (net-zero 2050) global warming emissions reduction goal.

These findings highlight the importance of acting now because of the long lifetimes of existing vessels in the next decade to prevent an overshoot of the 1.5°C emissions reduction goal. We know from global energy system analysis that immediate climate action can limit global warming to well below 2°C.^60^ These quick climate actions should start now to ensure a green transition of the hard-to-abate sector^61^ that is international shipping.

With the indicated levels of carbon pricing, fuel savings and growth rates for upscaling green-fuel production capacities, a shipping industry in line with the Paris Agreement is theoretically achievable. However, it requires a lot to reach the indicated levels of the respective instrument for a green transition pathway, e.g., technological progress and fuel demand savings (further development and investments in green-fuel production capacities, alternative engines, renewable/clean energy, and fuel savings in general), regulatory changes (uniform WTW emissions accounting standards, long-term sustainable fuel directives, new contractual designs^42^), and policy actions (ambitious carbon-pricing pathways and uniform expectations across stakeholders to drive investments in green technologies). Thus, the focus of the future climate policy for the maritime industry should be on accelerating the upscaling of green fuels, incentivizing green fuels, and enabling fuel savings as fast as possible, especially because the expected emissions in the next ten years will make it hard to get into line with the Paris Agreement given the limited amount of green fuels available. Now is the time to start a holistic transformation of the maritime industry to pave the way for a sustainable maritime future.

## STAR★Methods

### Key resources table

**Table:** 

REAGENT or RESOURCE	SOURCE	IDENTIFIER
**Deposited data**		

GitHub repository with SEAMAPS code and data	This paper	https://github.com/SebastianFra/SEAMAPS

**Software and algorithms**		

Julia	The Julia Programming Language	https://julialang.org/

### Resource availability

#### Lead contact

Further information and requests for resources and reagents should be directed to and will be fulfilled by the lead contact Sebastian Franz (semfr@dtu.dk)

#### Material availability

This study did not generate new unique reagents.

### Method details

Our least-cost optimization model is called "SEAMAPS".^62^ SEAMAPS combines the transparency of emissions along the entire supply chain of green fuels, the constraints of upscaling green-fuel production capacities, representation of biomass availability, the emission reduction goals motivated by Paris Agreement narratives, decision modeling based on least-cost optimization, and the introduction of two dimensions of climate action (carbon-pricing and fuel savings) to understand the dynamics and challenges of climate mitigation. SEAMAPS is written in the mathematical programming language Julia and uses mixed-integer linear programming; its computing time for the analyzed set of scenarios is below 1 min (used solver: Gurobi), and all data and code are available as open source.^62^

In Figure 5, we show the modelling environment of SEAMAPS. It consists of multiple endogenous and exogenous data inputs.

The basic idea behind this model is least-cost optimization, which is used as the objective. The overall goal is to obtain the least-cost fueling options for the maritime industry. To achieve this, the objective function is minimized. The components of the objective function can be divided into two main parts, one which concerns all costs related to the fleet itself, including the investment costs for additional vessels, operations and maintenance costs. The second cost block is limited to fuel costs. The consumption of each vessel in the fleet is multiplied by the fuel costs (including fuel taxes, if any).

The objective function looks as follows:

(Equation 1) $$\min_{\partial ,\beta ,\theta ,\varepsilon }\sum_{s,y}\;\pi_{s}\;*{NB}_{s,y}+\gamma_{s}*{SS}_{s,y}+\pi_{s}+\sum_{s,f,y}F_{f,s,y}*\left(\vartheta_{f,y}+\mu_{f,y}\right)$$

Where $\pi_{s}$ is the investment expenditure for a new build (average) vessel of type s, ${NB}_{s,y}$ is new built ships of ship-type s in year y, $\gamma_{s}$ is the operation and maintenance cost for a vessel of type s, ${SS}_{s,y}$ is ship-stock of ship-type s in year, $F_{f,s,y}$ is the amount of fuel used per fuel-type f, ship-type s and year y, $\vartheta_{f,y}$ is the fuel cost per fuel type and year,and $\mu_{f,y}$ is the fuel tax added on top of fuel cost.

Additional constraints are added to adapt the future fuel mix to the future climate mitigation challenges of this hard to abate sector.

The most relevant constraints of the SEAMAPS model are described in the following:

#### Transport demand

(Equation 2) $$\forall_{sc,y}\;;\;D_{t,sc}=\sum_{s}{SS}_{s,y}*\rho_{s,y}*\beta_{sc,s}\;$$

This constraint limits the supply in the SEAMAPS model to the exogenous demand projections of the IMO^29^($D_{t,sc}$). We use an SSP1^48^-type demand. This ensures that supply and demand are matched and that there is no excess demand or supply in the model that could distort the results. It is important to note that the IMO demand has a strong influence on the results of the future fuel mix and that this variable might have to be replaced by endogenous demand projections in the future to create a more inherent modelling process. ${SS}_{s,y}$is the stock of ships s at year y, $\rho_{s,y}$ the average transport work of ship s in year y. $\beta_{sc,s}$ is a matrix relating the ship category (container, tanker, bulk, cargo, other) and the shiptype (ship category associated with a specific engine).

#### Fuel consumption

(Equation 3) $$\forall_{s,y}\;;\;\sum_{f}F_{f,s,y}*\;\alpha_{f,s}={SS}_{s,y}*\;\rho_{s,y}$$

The amount of fuel used by ships of type s in year y must be enough to satisfy the transport demand. The transport demand of the fleet of ships of type s is equal to the ship stock of that type (the number of ships of type s in the fleet) multiplied by the average transport work. The fuel consumption is calculated using the specific fuel consumption per fuel type and shiptype, $\alpha_{f,s}$. Any kind of fuel can be used to satisfy the demand, meaning that more than one fuel type can be used in the same year if the engine is a dual/multi-fuel engine.

#### Fuel availability

(Equation 4) $$\forall_{f,y}\;;\;\sum_{s}F_{f,s,y}\leq \omega_{f,y}$$

For all fuels and all years, the amount of fuel used for the whole shipping fleet cannot exceed the fuel available, which is represented by $\omega_{f,y}$.

#### Upscaling green-fuel production capacities

(Equation 5) $$\forall_{f,y}\;;\;\rho_{f,y}=\rho_{f,y-1}*\tau$$

For all fuels and all years, the available fuel $\rho_{f,y}$ is equal to the previous year's fuel availability multiplied by τ,which represents the expected yearly growth of green-fuel production capacities. This constraint is implemented in a way that the upscaling only starts once the model invests in the respective green fuel for the first time.In this study, we test different growth rates for the upscaling of green-fuel production capacities. Thus, we assume a conventional growth of 50% per year and an unconventional growth of 126% a year over 30 years (see Table 1). These two slopes have been motivated by diffusion speeds of historical solar PV growth and wind turbines for the case of conventional growth and the diffusion speed of US nuclear weapons and US aircraft during World War II for the case of unconventional growth.^40^

### Quantification and statistical analysis

This study did not use any specific tools.

## Data Availability

• All data have been deposited on our GitHub repository (https://github.com/SebastianFra/SEAMAPS) and are publicly available as of the date of publication. • All original code has been deposited on our GitHub repository (https://github.com/SebastianFra/SEAMAPS) and ist publicly available as of the date of publication • Any additional information required to reanalyze the data reported in this paper is available from the lead contact upon request.
