# Supplementary material for: Requirements for a maritime transition in line with the Paris Agreement
Source: iScience. 2022 Nov 18;25(12):105630. doi: 10.1016/j.isci.2022.105630 (PMC9730049; doi:10.1016/j.isci.2022.105630)
Supplement: Document S1. Figures S1–S9 and Tables S1–S18 [file mmc1.pdf]

iScience, Volume *25*

Supplemental information

Requirements for a maritime transition in line with the Paris Agreement

Sebastian Franz, Nicolas Campion, Sara Shapiro-Bengtsen, Rasmus Bramstoft, Dogan Keles, and Marie Münster

Requirements for a maritime transition in line with the Paris Agreement

Supplementary Information

Authors: Sebastian Franz[1,*], Nicolas Campion[1], Sara Shapiro-Bengtsen[1], Rasmus Bramstoft[1], Dogan Keles[1] , Marie Münster[1]
Affiliation:
[1] Technical University of Denmark, Department of Technology, Management and Economics, Energy Economics and System Analysis

[*] Corresponding Author: semfr@dtu.dk

Table of Content

1. Modelling environment 3
2. Fuels for the maritime industry 4
3. General Assumptions for the e-fuel modelling process..... 5
4. Fuel prices 6
5.Modelling of the Direct-Air-Capture(DAC) Fuels 9
6. Cost reduction of e-fuels..... 10
7. Fuel emissions..... 11
8. Grey and blue ammonia..... 12
9. Exogenous Biomass availability scenarios 13
10. Competing demand..... 14
11. Demand projections..... 16
12. Shipping stock & engines & total cost of ownership 17
13. Sensitivity analysis 19
Bibliography 22

1. Modelling environment

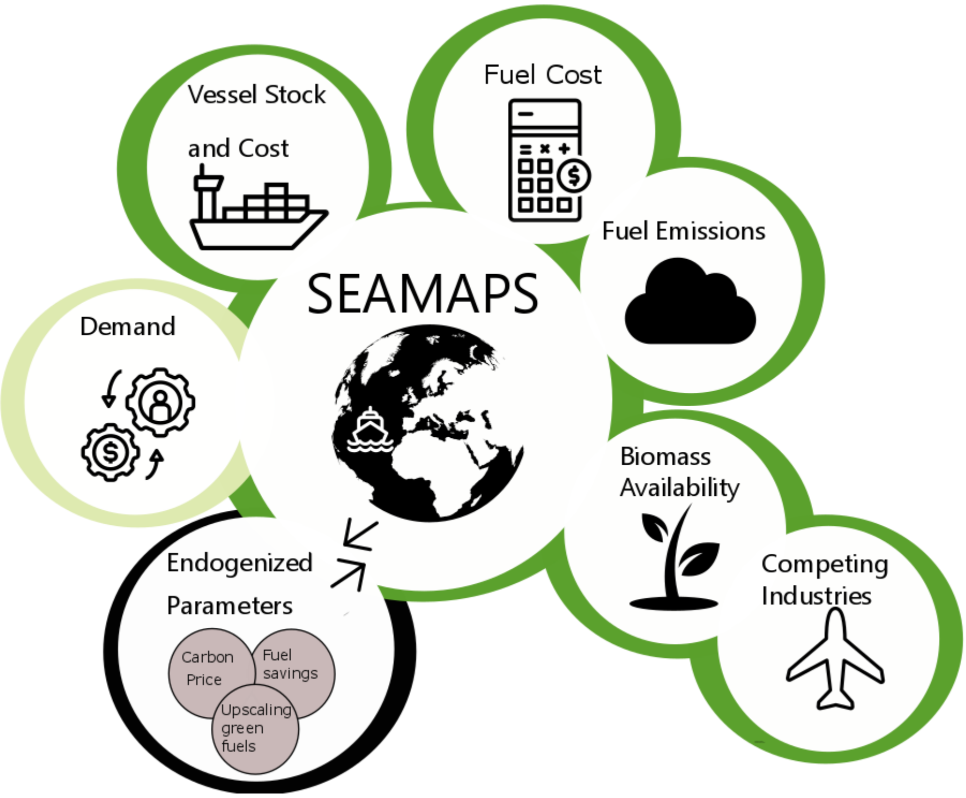

Figure S1: SEAMAPS modelling approach related to STAR Methods

The modeling environment of this work can be split into several parts. All modelling areas ultimately are being integrated in the SEAMAPS model. SEAMAPS is a least-cost optimization model for the maritime industry featuring detailed and novel data on life-cycle emissions and cost of green fuels, biomass availability and competing industries, total cost of ownership for vessels and engines as well as endogenized parameters to drive climate mitigation of the maritime industry. In the following the different modelling areas, data and assumptions are being discussed and derived.

2. Fuels for the maritime industry

Abbreviation	Full Name	Category	Production Method
VLSFO/HFOsc	Very Low Sulphur Fuel Oil /Heavy fuel oil	Fossil Fuel	Conventional
MDO	Marine Diesel Oil	Fossil Fuel	Conventional
MGO	Marine Gas Oil	Fossil Fuel	Conventional
LNG	Liquified Natural Gas	Fossil Fuel	Conventional
MeOH-grey	Methanol	Fossil Fuel	Conventional using Natural Gas
MeOH-ebio	Bio-e-Methanol	Green Fuel	Biomass-to-Methanol via thermochemical conversion boosted with electrolytic hydrogen[1]
MeOH-PS	E-Methanol	Green Fuel	CO_2 hydrogenation using hydrogen from electrolysis and renewable CO_2 (according to IRENA terminology) from Point-Source from biomass-fired plant
MeOH-DAC	E-Methanol	Green Fuel	CO_2 hydrogenation using hydrogen from electrolysis and renewable CO_2 (according to IRENA terminology from Direct Air Capture
NH3-grey	Grey Ammonia	Fossil Fuel	Conventional using Natural Gas
NH3-blue	Blue Ammonia	Blue Fuel	Haber–Bosch process using Natural Gas (steam-methane reformation) & Carbon Capture Storage
NH3-green	Green Ammonia	Green Fuel	Haber–Bosch process using hydrogen from electrolysis*
LBG	Liquified Biogas (bio-methane)	Green Fuel	Anaerobic digestion of biomass subsequently upgraded to biomethane and liquified

Table S1: Description of all analyzed fueling options, including their category and production method (for more details, see Nami et. al. 2021[1] and Franz et. al. 2021[2] and SI section 1.2) related to STAR Methods

[1] Using 100% of solar and wind electricity

3. General Assumptions for the e-fuel modelling process

The derivation of e-fuels in this study features some life-cycle elements. To increase transparency in this regard we show the main assumptions behind the life-cycle elements of GHG emissions for the analysed e-fuels in table S2 and S3.

In the main text we refer to these assumptions when we talk about upstream emission for fuel infrastructure. These are all assumed to decline linearly over time to reach zero in 2050.

Process	Value	Unit	Source	Note
Methanol plant	18.5	$kgCO_{2e}$/(kg MeOH/h)/year	Ecoinvent[a]	Plant capacity 2700 t/day. 30 year lifetime.
H_2 storage	0.006	$kgCO_{2e}$/kg H2/year	[3]	Compressed hydrogen storage 330 m3/tank. Ecoinvent processes for material use. Assumed lifetime 30 years.
Electrolyser AEC	2.29	$kgCO_{2e}$/kW/ year	[4]	Assumed 8000 load hours per year.
Electrolyser SOEC	2.73	$kgCO_{2e}$/kW/ year	[4]	Assumed 8000 load hours per year.
Battery	1.57	$kgCO_{2e}$/kWh/ year	Ecoinvent[b]	Assumed specific energy density: 0.265 kWh/kg
Ammonia plant	18.5	$kgCO_{2e}$/(kg NH_3/h)/year	Ecoinvent[a]	As no data was found on ammonia plants the same data is used as for methanol plant infrastructure.
Air separation unit	4.81	$kgCO_{2e}$/kg N_2/h/year	Ecoinvent[c]	Cryogenic air separation.
Gasifier	4.09	$kgCO_{2e}$/(kg syngas/h)/year	Ecoinvent[d]	Synthetic gas production from wood. Fluidized bed gasifier.
Carbon capture	5.68	gCO_{2e}/ kg CO_2 captured	[5]	Captured from ambient CO_2. Emissions for building point source CO_2 capture are assumed to be 5% of that of building infrastructure for capturing ambient CO_2 (own assumption)

All Ecoinvent processes taken from the consequential database v. 3.7.1. , specific processes as follows:
[a]methanol factory construction – GLO, [b]battery cell production, Li-ion - RoW, [c]air separation, cryogenic - RER/RoW, [d]synthetic gas production, from wood, at fluidized bed gasifier – RoW.

Table S2: Overview of assumptions for GHG emissions associated with infrastructure for e-fuels, Related to figure 1

Technology	kg CO2e/kW/year	Source	Note
Onshore wind	55	Ecoinvent[a]	Data from 2008. Reference for wind turbines with a capacity of larger than 3 MW
Solar PV	91	Ecoinvent[b]	

All Ecoinvent processes taken from the consequential database v. 3.7.1. , specific processes as follows:
[a]wind turbine construction, 4.5MW, onshore - GLO, [b]photovoltaic plant construction, 570kWp, multi-Si, on open ground - GLO.

Table S3: Overview of assumptions for GHG emissions associated with electricity-generation, Related to figure 1

4. Fuel prices

Fuel prices are determined with two different methods:

- If the fuel market price is available (e.g., for fossil fuels), the price is derived from real market data and IEA predictions[6,1]. By convention, prices are taken in Rotterdam.
- If the fuel is not on the market already (e.g., electro-fuels), the price is derived from the estimated fuel production cost using the method presented in figure S2. The assumptions and methodology used to estimate the fuel production cost are taken from [1].

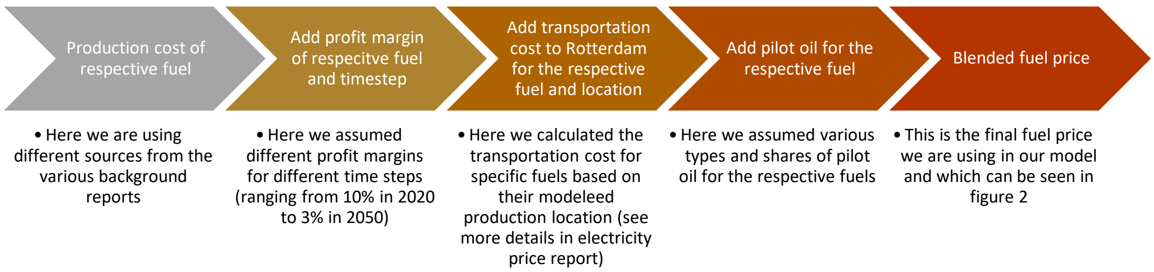

Figure S2: Fuel price derivation process, Related to figure 1

Fuel market prices in Rotterdam are used as references. On top of that, the price of pilot fuel is added to the main fuel. For more details see[2,6].

	**Source**	**Original fuel price* [€2019/GJ]**				**Blended fuel price if different from original [€2019/GJ]**			
		**2020**	**2030**	**2040**	**2050**	**2020**	**2030**	**2040**	**2050**
HFOsc/ VLSFO#	**CE Economics**[6]	10.2	9.0	8.6	8.1	-	-	-	-
MDO¤	**CE Economics**[6]	10.9	9.6	9.2	8.7	-	-	-	-
MGO	**CE Economics**[6]	10.9	9.6	9.2	8.7	-	-	-	-
LNG	**CE Economics**[6]	4.3	3.1	3.1	3.2	4.4	3.2	3.5	3.5
MeOH-Grey	**CE Economics**[6]	9.7	7.4	7.5	7.6	9.7	7.5	8.5	8.4
NH3-grey	**CE Economics**[6]	10.7	8.2	8.2	8.3	10.7	8.2	9.2	9.1
NH3-blue	**DTU Energy**[1]	30.0	24.0	22.6	21.1	29.0	23.3	22.9	21.2
LBG	**DTU Energy**[1]	20.3	28.2	34.2	46.6	20.1	27.9	34.1	46.2

* Market price in Rotterdam. Future price estimated using the Sustainable Development Scenario from IEA [see "FUEL PRICES AND LEARNING RATES Contribution" by Copenhagen Economics]

To simplify the problem, it is assumed that HFO and scrubber has the same price as Very Low Sulphur Oil

¤ Not found on market data assumed equal to be equal to MGO price

Table S4: Blended fuel prices derived from market prices, Related to figure 1

The price of fuels that cannot be derived from existing market prices from[6] are thus calculated by adding production cost, transport cost to Rotterdam (reference price location), and profit margin. For more details see[2].

Production cost

The greenest way of producing electro fuel is to only use local renewable power production. For more details see[2].

Summary of the production cost of e-fuels used in the model is shown in table S5:

	**Selected production site**				**Production cost [€2019/GJ fuel]**			
	**2020**	**2030**	**2040**	**2050**	**2020**	**2030**	**2040**	**2050**
MeOH-e-bio	Dakhla	Dakhla	Dakhla	Dakhla	38.4	30.8	28.3	25.7
MeOH-PS	Dakhla	Dakhla	Dakhla	Arica	48.5	38.7	33.4	28.0
MeOH-DAC	Dakhla	Dakhla	Dakhla	Arica	57.8	46.4	39.8	33.2

NH3-green	Dakhla	Dakhla	Dakhla	Arica	42.7	33.6	28.1	22.6

Table S5: Selected production sites and associated cost in the behind-the-meter configuration [see the report "MarE-Fuel: Energy efficiencies in synthesizing green fuels and their expected cost" for more details]. Dakhla: West Sahara, very good wind and solar potential. Arica: North Chile, excellent solar potential, low wind potential, Related to figure 1.

Transportation cost

In order to compare all fuels on a similar basis, it was assumed that all fuels would be shipped to Rotterdam. The production costs between the production site of each fuel and Rotterdam were calculated using the following simple equation:

$$Transport\ cost_{s,f}[€2019/GJ] = \frac{cargo{-}cost\ per\ tonne_{s}\ (based\ on\ rotation\ duration\ between\ O\&Ds)}{calorific\ value\ of\ fuel_{s,f}} \quad (1)$$

Where:

s= production site (in our approach Dakhla, Arica, Esbjerg, and Ceduna, for more details see [7])

f= type of fuel (we only calculated transport cost for electro-fuels as the other fuels are already market price-based fuels

The variable "cargo-cost per tonne" for the respective production site is based on the assumed rotation time, which consists of loading, transit, unloading and return. This rotation duration of the different origins and destinations (O&Ds) is then linked to the average monthly cost of the vessels (for more details see[8]). For more details see[2].

Obtained transport costs applied to the original fuel cost are presented in Table S4.

Transportation cost to Rotterdam [€2019/GJ fuel]	Arica	Dakhla	Ceduna	Esbjerg
Methanol	0.76	0.29	1.09	0.06
Ammonia	1.08	0.31	1.54	0.01

Table S6: Transportation cost to Rotterdam, Related to figure 1.

Profit margin

The profit margin added to fuel production costs is assumed to be relatively high in 2020 and to decrease over time due to increasing competition. The values used for the price calculation are shown in Table S7. The profit margin is applied before including transportation costs. For more details see[2].

	**2020**	**2030**	**2040**	**2050**
Profit margin on fuel production cost	10%	7%	5%	3%

Table S7: Profit margin added to the fuel production cost, Related to figure 1

Blended fuel prices

The resulting prices for blended electro-fuels, including transport from the selected production site to Rotterdam, are shown in table S8. For more details see[2].

	**“Blended” fuel price Behind meter [€2019/GJ fuel]**			
	**2020**	**2030**	**2040**	**2050**
MeOH-e-bio	41.3	32.6	30.5	26.8
MeOH-PS	52.0	40.7	35.6	29.5
MeOH-DAC	61.8	48.6	42.0	34.5
NH3-green	45.9	35.0	29.9	24.5

Table S8: Blended electro-fuel price in Rotterdam when produced off-grid and partially on-grid, Related to figure 1

5.Modelling of the Direct-Air-Capture(DAC) Fuels

For the DAC fuel modelled in this study we used a set of assumptions which can be seen in TableS9.

	2020	2030	2050
Power consumption [kWe/kg CO_2 captured]	0.5	0.47	0.43
Specific investment [€2019 / (kg CO_2 captured/h)]	7000	6000	4000
Fixed operation and maintenance [€2019 / (kg CO_2 captured/h) /y]	350	300	200
Variable operation and maintenance [€2019 / kg CO_2 captured]	0	0	0
Projected cost of resulting methanol (€/t)	1053	811	558

Table S9: Techno-economic assumptions[9] *used in the model for DAC system, Related to figure 1*

6. Cost reduction of e-fuels

The cost reduction of e-fuels are calculated within the Optiplant[7] optimization model. The underlying assumptions can be found in the following table and result in decreasing costs for e-fuels over time.

	**Investment** (€2019/Capacity[10])			**Fixed cost** (€2019/Capacity[10]/y)			**Fuel buying price**[11] (€2019/kg output)			**Lifetime** Years		
**Type of unit**	**2020**	**2030**	**2050**	**2020**	**2030**	**2050**	**2020**	**2030**	**2050**	**2020**	**2030**	**2050**
**CO2 capture DAC**[12]	7000	6000	4000	350	300	200	0	0	0	20	20	20
**CO2 capture PS**[12]	2700	2300	1600	81	69	48	0.0025	0.0025	0.0025	20	20	20
**MeOH plant CCU - AEC**	4168	4168	4168	618	618	618	0.027	0.027	0.027	25	25	25
**Biomass supply**[12]	-	-	-	-	-	-	0.125	0.136	0.157	-	-	-
**Bio-eMeOH plant - AEC**	4085	4085	4085	578	578	578	-	-	-	25	25	25
**Bio-eMeOH plant - SOEC**	4085	4085	4085	578	578	578	-	-	-	25	25	25
**NH3 plant + ASU - AEC**	4285	4285	4285	601	601	601	-	-	-	25	25	25
**NH3 plant + ASU - SOEC**	4285	4285	4285	601	601	601	-	-	-	25	25	25
**Desalination plant**[13]	26	26	26	-	-	-	0.0003	0.0003	0.0003	20	20	20
**Electrolyser AEC**	55371	41435	17526	1107	829	351	-	-	-	25[14]	25[14]	25[14]
**Electrolyser SOEC**	153351	57744	19703	3183	835	556	-	-	-	25[14]	25[14]	25[14]

Table S10: Economic assumptions used in the optimization model, Related to figure 3

7. Fuel emissions

In this study, we defined a baseline scenario and built two carbon pricing pathways upon this baseline scenario. In our baseline scenario, we assume a medium availability of biomass for the maritime industry (for more details, see SI section 1.4). Furthermore, our baseline scenario assumes an islanded production of green fuels by only using electricity from its own renewable electricity production.

Table S11 shows the WTT(Well-To-Tank), TTW(Tank-to-Wake), and WTW(Well-to-Wake) emissions of all considered fuels in 2020. Figure S3 shows the decrease in WTW emissions for the modelled green fuels. The WTT emissions coming from the use of pilot fuel are accounted for. Infrastructure emissions are assumed to drop to zero in 2050. This is a strong assumption that all infrastructure production processes (e.g., mining) will be CO_2 neutral in 2050.

Fuels	WTT	TTW	WTW
VLSFO/HFO	13	77	90
MDO	12	75	87
MGO	16	75	91
LNG	14	78	92
MeOH-grey	22	70	92
MeOH -e-bio	7	4	11
MeOH -PS	14	4	18
MeOH -DAC	15	4	19
NH3-grey	136	4	141
NH3-blue	50	4	54
NH3-green	14	4	18
LBG	28	24	52

Table S11: Average values of emissions by fuel (include pilot fuel) in kg CO2e/GJ in 2020 using Global Warming Potential(GWP) of 100 years, Related to figure 3

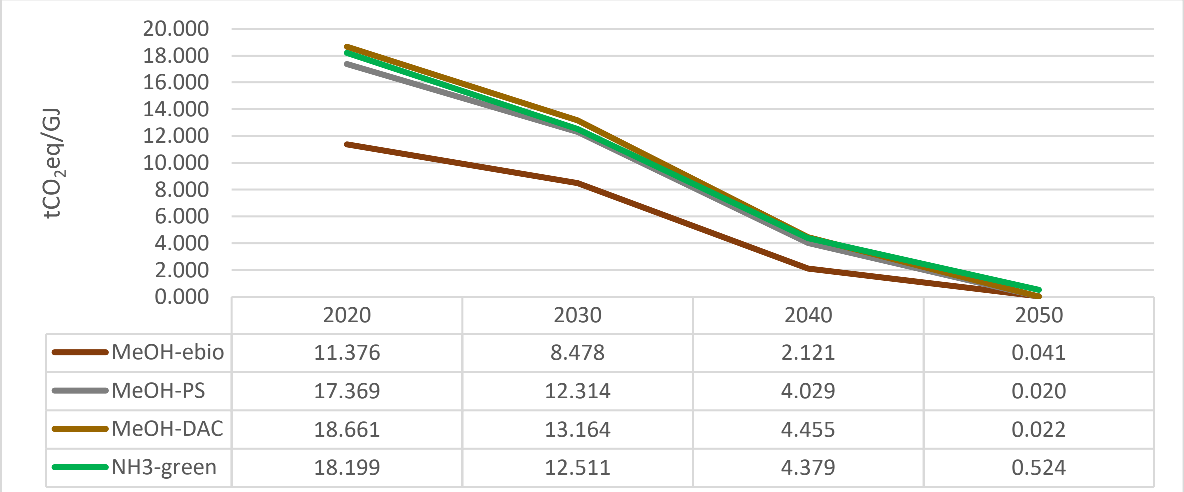

Figure S3: Emission intensity of producing e-fuels from a full lifecycle perspective when produced off-grid, Related to figure 3

8. Grey and blue ammonia

A large number of studies have been carried out on greenhouse gas emissions from grey and blue ammonia. However, these studies usually use different assumptions and it is a challenge to adjust the assumptions for the accounts of GHG emissions from blue and grey ammonia while using a wide range of literature. In order to make a fair comparison between blue and grey ammonia, emissions are calculated using the same input data.

Grey ammonia emissions include emissions associated with the production process and methane leakage that occurs during the transport and extraction of natural gas (upstream emissions). Upstream emissions are difficult to quantify and the range of uncertainty is extensive and highly dependent on the location of gas extraction. The committee on climate change reported the range of natural gas upstream emissions between 15 and 70 kg of CO_2e /MWh of gas (LNG) [15]. Natural gas consumption in the ammonia production process is 0.71 kg gas/kg NH_3[16], and process emissions used are 2.4 kg CO_2/kg NH_3 produced (values ranging between 1.6 to 2.7 for main regions, up to 4 kg CO_2/kg NH_3 when ammonia is produced from coal)[17].

Blue ammonia emissions are estimated using the grey ammonia emissions with the use of Carbon Capture and Storage (CCS) technology on the ammonia production site. The upstream emissions due to methane leakage during natural gas production are unchanged. CCS system applied on the ammonia plant is assumed to have an (optimistic) efficiency of 90% of CO_2 captured[18], meaning that process emissions are reduced to 0.24 kg CO_2/kg NH_3.

Calculations and assumptions used to estimate blue and grey ammonia emissions are summarized in table S12:

GHGs emissions	**Minimal**	**Base case**	**Maximal**
**Grey ammonia**			
Natural gas consumption ammonia process [t gas/tNH_3]	0.72	0.72	0.72
Grey ammonia upstream emissions (from natural gas) [tCO_2e/tNH_3]	0.15	0.425	0.70
Grey ammonia process emissions [tCO_2/tNH_3]	1.6	2.4	2.7
**Total grey ammonia emissions [kg CO_2e / GJ NH_3]**	**94.0**	**151.8**	**182.6**
**Blue ammonia**			
Carbon Capture efficiency [% CO_2 captured]	90%	90%	90%
Blue ammonia process emissions [tCO_2/tNH_3]	0.16	0.24	0.27
Blue ammonia upstream emissions (from natural gas) [t CO_2e/t NH_3]	0.15	0.42	0.7
**Total blue ammonia emissions [kg CO_2e/GJ NH_3]**	**16.6**	**35.6**	**52.0**

Using the LHV of 18.6 MJ/kg for NH_3 and 50MJ/kg for LNG

Table S12: Grey vs Blue Ammonia emission calculations, Related to figure 3

9. Exogenous Biomass availability scenarios

The production of biofuels for shipping must be based on cost-effective, sustainable resources. The biomass considered is limited to residual biomass. The annual technical resource potential, taking into account environmental sustainability aspects, is shown in Table S13, assuming that it does not change over time. For more details see Franz et.al[2].

For simplicity, we have in this study considered the residual biomass potential to be carbon neutral. We have hence omitted upstream emissions related to e.g. biomass collection, drying, pelleting and transport here, as it varies substantially based on local conditions. Applying a life-cycle perspective not only to the indirect emissions of fuel infrastructure but also to the biomass "production" process could be a topic for further research.

Resource	Low	High	Source
Crop residues	15 EJ	70 EJ	[19]
Forestry residues	13 EJ	15 EJ	[20]
Black liquor	2 EJ	2 EJ	[21]
Manure	0 EJ*	13 EJ**	[22,23]
Organic waste	0 EJ*	2 EJ**	[22,23]

*Assumed to be zero in the low availability scenario

**listed as biogas potential

Table S13: Overview of annual technical resource availability, Related to figure 2.

10. Competing demand

Biogenic carbon is in high demand for energy and non-energy purposes. In this study, competing demand has been limited to power generation, air and road freight, and petrochemicals (plastics) (see Figure S4). The expected demand for these sectors is thus subtracted from what is available for marine fuel production.

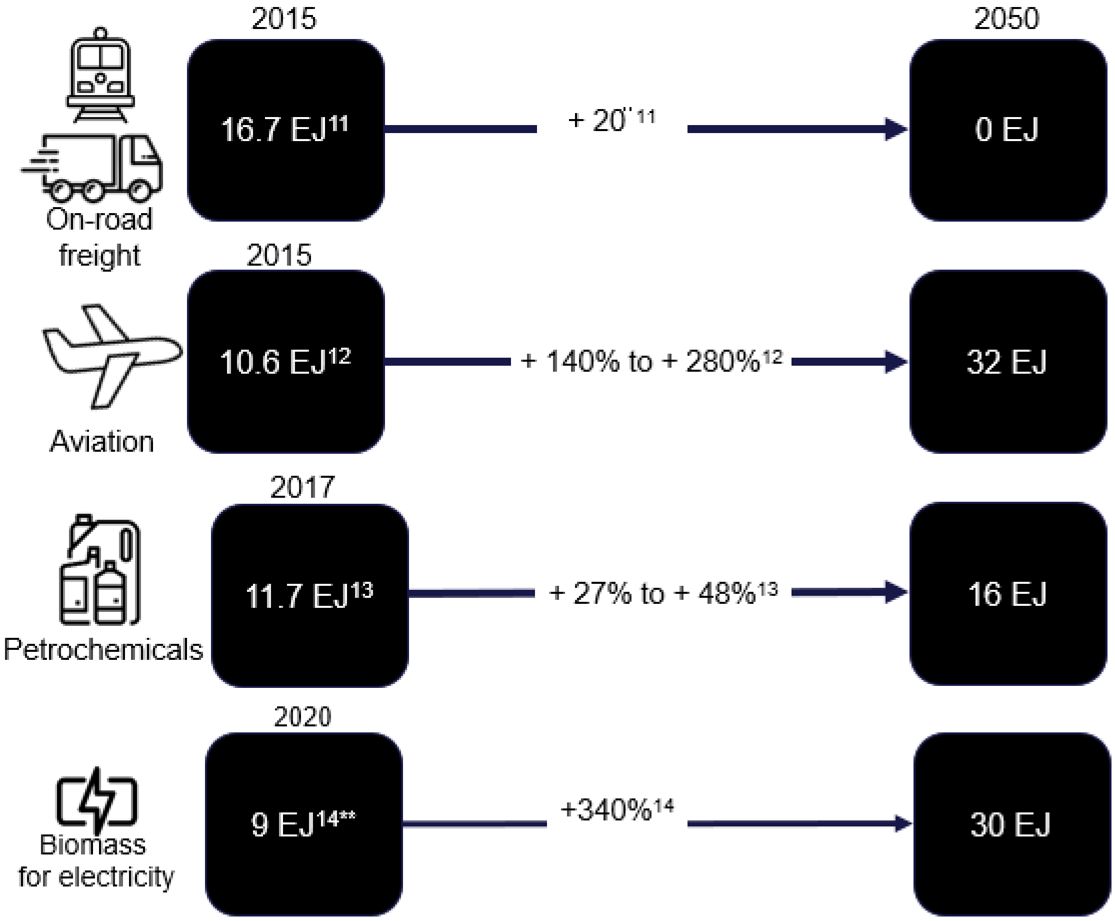

Figure S4: Competing Demand for Biogenic Carbon For more details see[2]. Data on competing industries by: Freight[24], Aviation[25], Petrochemicals[26], Electricity[27]. For more details, see Franz et. al. 2021[2], Related to figure 2

Our baseline scenario features a medium biomass availability and medium competing fuel demand. An optimistic scenario with some availability in the short term, see figure S5.

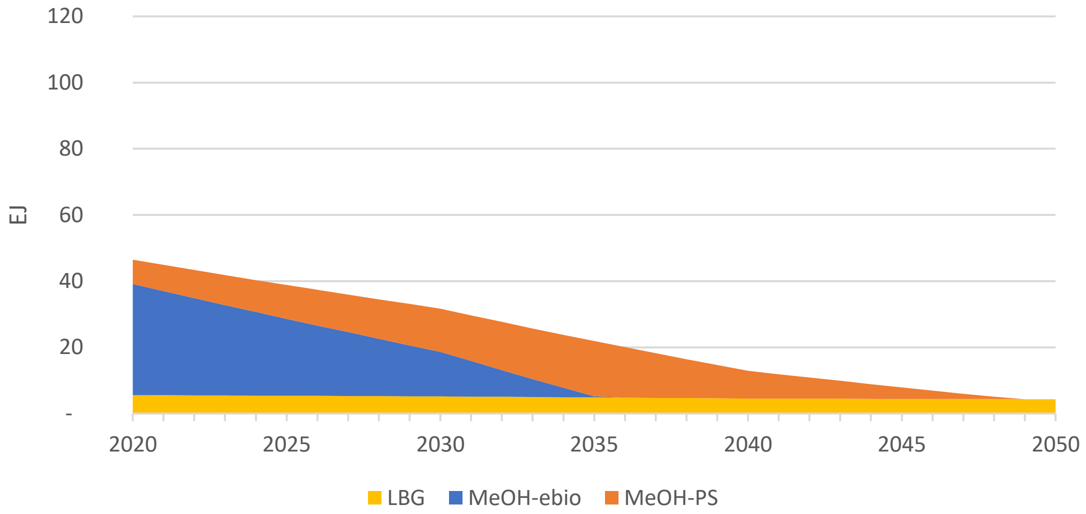

Figure S5: Medium biomass availability medium competing demand, MET-e-bio, Related to figure 2

11. Demand projections

In this work, exogenous demand projections from the IMO were used[8]. In our scenarios we have used the SSP1-type scenario throughout our analysis[28]. The underlying demand projections can be seen in Figure S6.

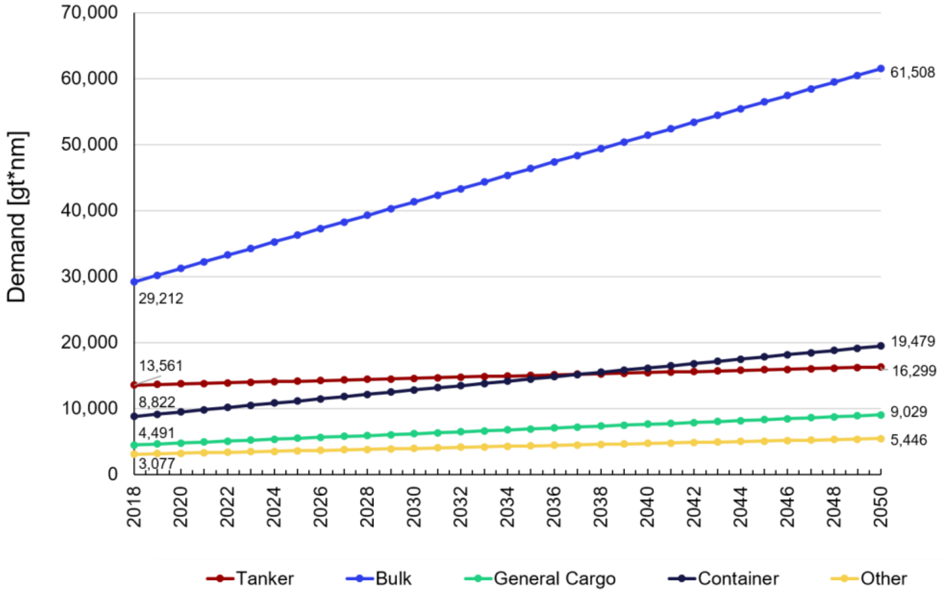

Figure S6: Ship Demand Projections for an SSP1 type scenario [8], Related to figure 4

12. Shipping stock & engines & total cost of ownership

Another essential input data for our least-cost optimization model are the ship and engine stock and the total cost of ownership of the respective ships.

In Table S14 we show the available engine-types with their first year of availability and the respective handled fuels[29,30]. We assumed the ships to be able to switch between fuels within a respective engine type without retrofitting cost.

		Fuels handled without additional retrofitting cost				
Engine type	**First year available**	**VLSFO/HFOsc**	**MDO/MGO**	**LNG/LBG**	**MeOH**	**NH3**
**ME-C**	2020	✓	✓	✗	✗	✗
**ME-GI**	2020	✓	✓	✓	✗	✗
**ME-LGIm**	2020	✓	✓	✗	✓	✗
**ME-LGIa**	2025	✓	✓	✗	✗	✓
**ME-LGImulti**	2031	✓	✓	✗	✓	✓
**ME-GImulti**	2031	✓	✓	✓	✓	✓

Table S14: Engine-Types and respective handled fuels, Related to figure 4

The CapEx of the average ships used in the model is calculated using cost estimates for existing fuel oil ships and expected added cost to handle a new type of fuel.

The cost estimates for existing fuel oil ships can be seen in table S15. For more details see[31].

**Engine size [MW]**	6	14	50
**Ship size [TEU]**	1200	2500	24000
**Vessel CapEx [MUSD]**	**18**	**28**	**150**

Table S15: Input data for CapEx calculations (see "MarE-Fuel: Total Cost of Ownership" for more details), Related to figure 4

The costs are then converted to €2019 and linearly interpolated to match the engine power of the average vessels used in the model.

Additional costs are added depending on engine type and fuel handling. These costs include the need for different fuel supply systems, tanks depending on fuel type and engines. Vessels operating on ammonia must also be equipped with water curtain systems and spill containment systems.

The additional CapEx estimates are presented in table S16.

Main fuel	Engine type	6 MW engine	14 MW engine	50 MW engine
Fuel oil	**ME-C**	0	0	0
LNG	**ME-GI**	5	8	13
Methanol	**ME-LGIm**	3	4.5	8
Ammonia	**ME-LGIa**	4	6	10

Table S16: Additional vessel CapEx estimates depending on the engine type and size in MUSD (see [31] for more details), Related to figure 4

The final CapEx for the respective ship-type, which has been used for this study, can be seen in Table S17. For more details see[2].

**Ship category**		**Other**	**Tanker**	**General Cargo**	**Bulk**	**Container**
**Average engine power [MW]**		2	5	5	8	28
**Main fuel**	**Engine type**	**Total CapEx new built vessel used in the model [M€2019]**				
Fuel oil	**ME-C**	5.6	15.0	15.0	18.8	70.3
LNG	**ME-GI**	7.5	18.9	18.9	24.4	79.7
Methanol	**ME-LGIm**	6.7	17.4	17.4	22.2	75.8
Ammonia	**ME-LGIa**	6.9	18.4	18.4	23.3	77.4
All fuels but LNG	**ME-LGImulti**	7.0	18.5	18.5	23.5	77.9
All fuels	**ME-GImulti**	8.0	21.0	21.0	26.8	83.2

Table S17: Additional vessel CapEx estimates depending on the engine type and size in M€2019, Related to figure 4

For operation and maintenance costs, we assumed the following costs: Crew salaries, supplies, maintenance, insurance, management fees, dry dock, and port fees. It is assumed that the costs for all types of fuels are similar and only differ depending on the size of the vessel. [29,31]

The O&M cost data per ship type and engine used in the model can be found in table S18.

**Ship category**	**Other**	**Tanker**	**General Cargo**	**Bulk**	**Container**
Average engine power [MW]	2	5	5	8	28
**O&M cost [M€2019/y]**	**0.9**	**2.1**	**2.1**	**2.5**	**3.3**

Table S18: O&M cost per ship type and engine type in M€2019, Related to figure 4

13. Sensitivity analysis

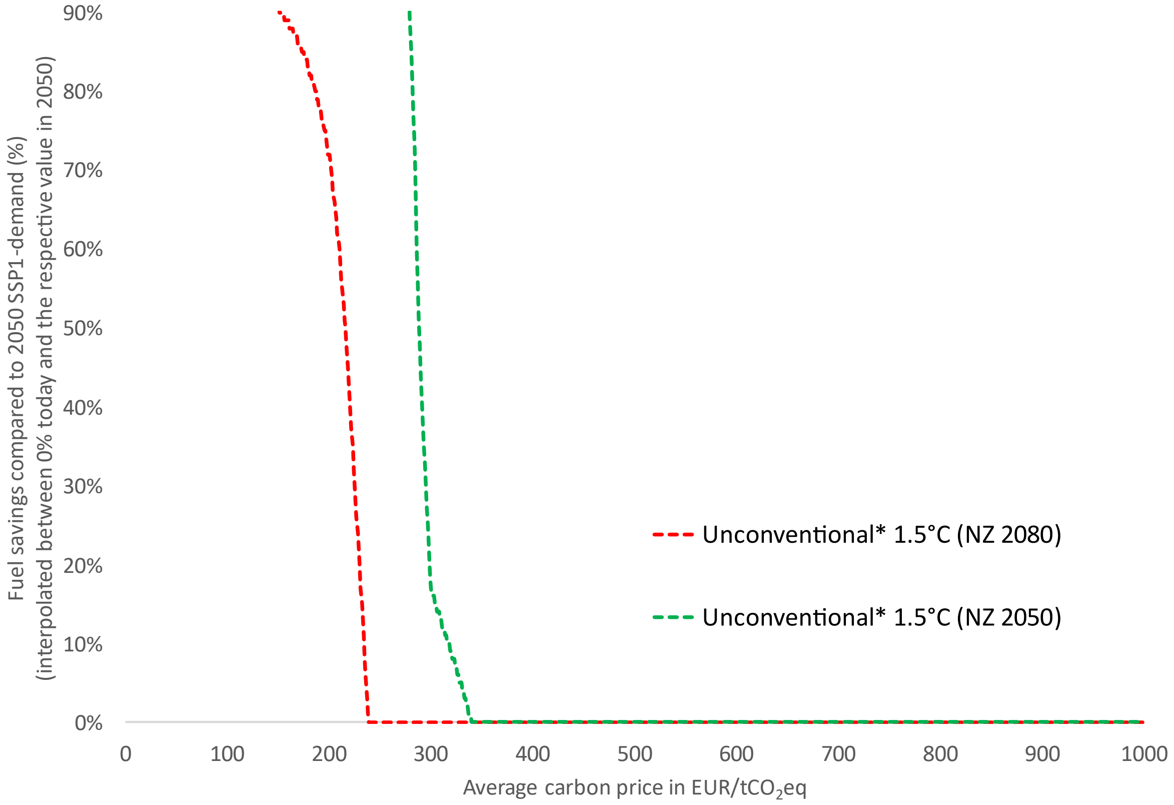

Figure S7: Solution Space to reach Paris Agreement goals with unconventional growth of green fuel production capacities, Related to figure 4

We performed a few sensitivities analyses on the most important constraints and assumptions to account for uncertainties that can be seen in the limitations section of the manuscript.

In addition to this we also ran the results with the unconventional growth rate of 126% of annual green fuel production capacity. The results can be seen in figure S7.
One can identify that with significantly higher growth rates for green fuels and thus faster fuel transition and higher green fuel availability the challenges towards climate mitigation are significantly lower then with the conventional growth rates (see figure 5 in the manuscript)

We choose the baseline scenario “CO2-tax Progressive” (for details, see table 1 in the main text).
Sensitivity Parameter:

- Costs for Green Fuels (see Figure S10 & S11 “Green Push - costs” (Reduction of Green fuel cost by 25% on average) and “Green Delay - costs” (Increase of Green fuel cost by 25% on average))
- Emissions for Green Fuels (see Figure S10 & S11 “Green Push - emissions” (Reduction of Green fuel cost by 25% on average) and “Green Delay - emissions” (Increase of Green fuel cost by 25% on average))

- Not using Upstream Emissions (see Figure S10 & S11 “No Upstream)

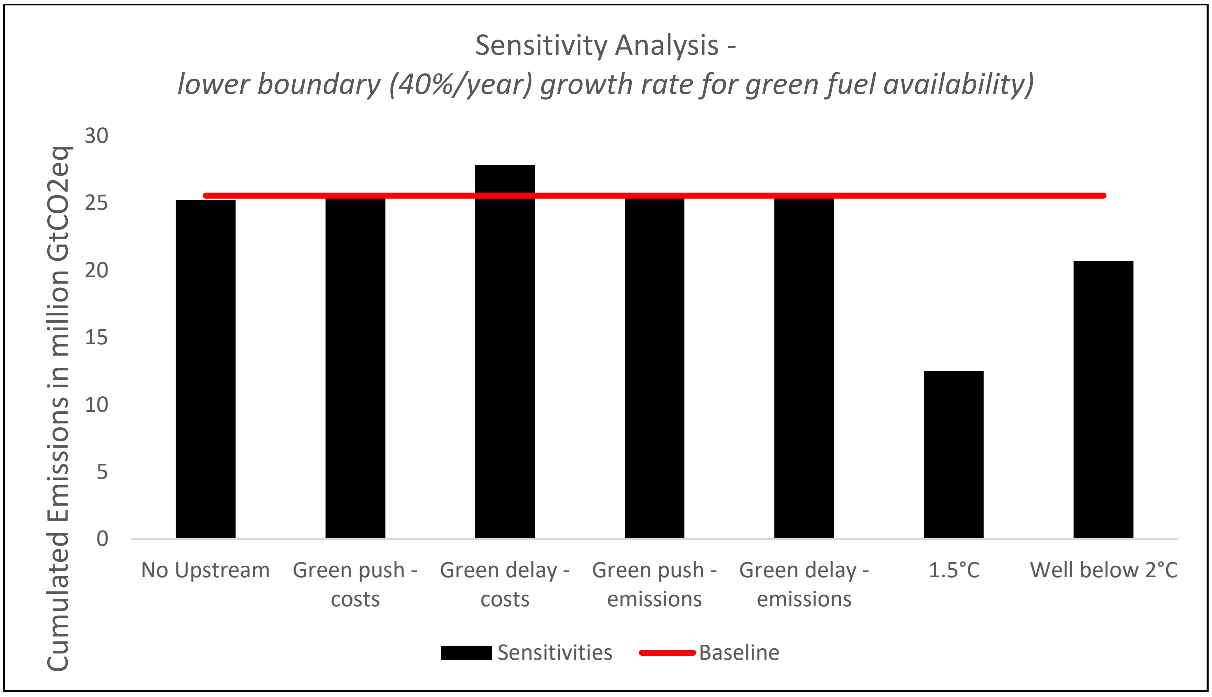

Figure S8: Sensitivities for the Baseline Scenario with a Progressing Carbon Pricing Scenario of 250EUR/tCO_2eq on average no fuel savings and lower boundary (40%/year) growth of green fuel availability per year, Related to figure 5

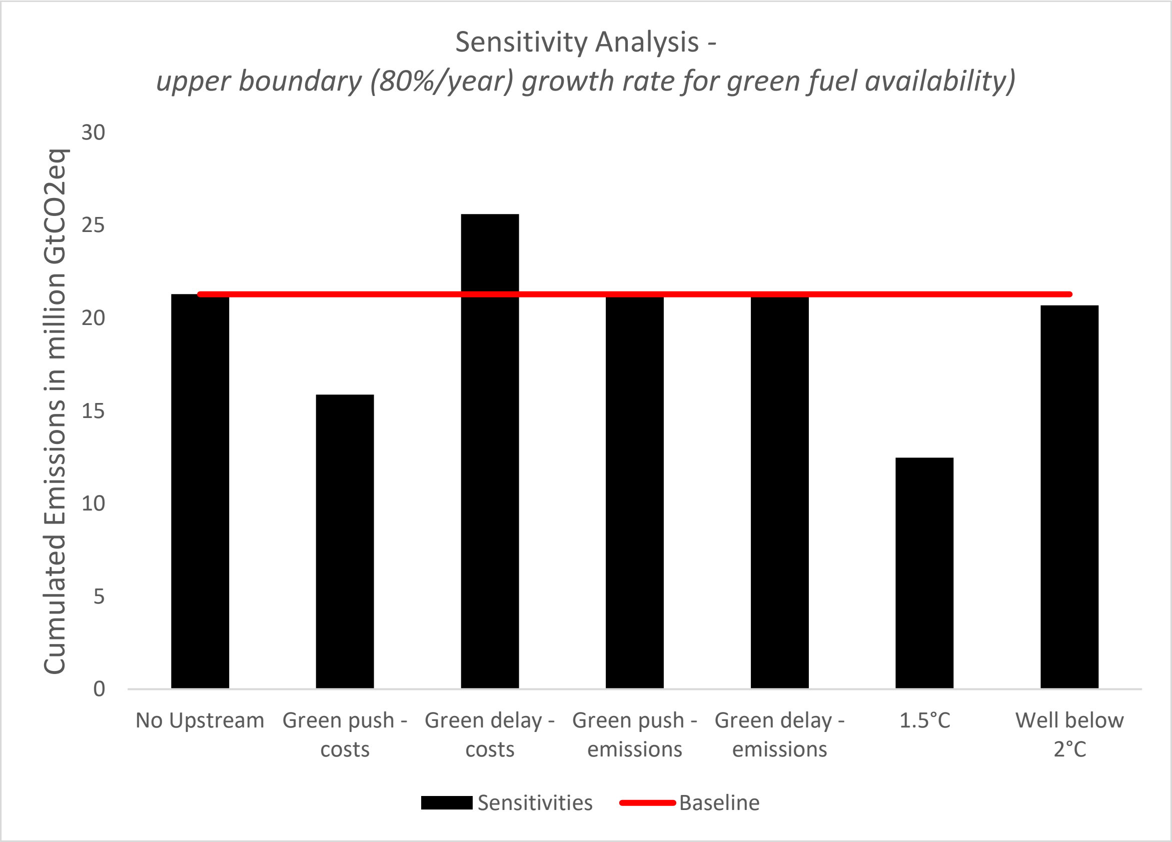

Figure S9: Sensitivities for the Baseline Scenario with a Progressing Carbon Pricing Scenario of 250EUR/tCO_2eq on average no fuel savings and upper boundary (80%/year) growth of green fuel availability per, Related to figure 5

Bibliography

1. Nami, H., Butera, G., Campion, N., Frandsen, H. L. & Hendriksen, P. V. *MarE-fuel: Energy efficiencies in synthesising green fuels and their expected cost*. (2021).
2. Franz, S., Campion, N., Shapiro-Bengtsen, S., Backer, M. & Münster, M. *MarE-Fuel: ROADMAP for sustainable maritime fuels*. (2021).
3. Deutz, S. & Bardow, A. Life-cycle assessment of an industrial direct air capture process based on temperature–vacuum swing adsorption. *Nat Energy* **6**, 203–213 (2021).
4. Brynolf, S., Kuvalekar, S. & Andersson, K. Life cycle assessment of methanol and dimethyl ether (DME) as marine fuels. (2014).
5. Brynolf, S., Fridell, E. & Andersson, K. Environmental assessment of marine fuels: liquefied natural gas, liquefied biogas, methanol and bio-methanol. *J Clean Prod* **74**, 86–95 (2014).
6. Copenhagen Economics. *CO2-Taxes, Fuel Prices and Learning Rates*. (2021).
7. Campion, N., Backer, M., Swisher, P. & Münster, M. *MarE-fuel: LCOE and optimal electricity supply strategies for P2X plants*. (2021).
8. IMO. *Fourth IMO GHG Study 2020 Full Report*. (2021).
9. Danish Energy Agency. *Technology Data for Industrial Process Heat*. (2020).
10. DTU Wind & World Bank. *Global Wind Atlas*.
11. UN Peacekeeping. *United Nations Mission for the Referendum in Western Sahara*. (2021).
12. Pfenninger, S. & Staffell, I. Long-term patterns of European PV output using 30 years of validated hourly reanalysis and satellite data. *Energy* **114**, 1251–1265 (2016).
13. Inflation Tool. *Inflation timeline in the eurozone*.
14. Koivisto, M. *et al.* Using time series simulation tools for assessing the effects of variable renewable energy generation on power and energy systems. *WIREs Energy and Environment* **8**, (2019).
15. Bourne, S. The future of fuel: The future of hydrogen. *Fuel Cells Bulletin* **2012**, 12–15 (2012).
16. Bellotti, D., Rivarolo, M., Magistri, L. & Massardo, A. F. Feasibility study of methanol production plant from hydrogen and captured carbon dioxide. *Journal of CO2 Utilization* **21**, 132–138 (2017).
17. Al-Breiki, M. & Bicer, Y. Investigating the technical feasibility of various energy carriers for alternative and sustainable overseas energy transport scenarios. *Energy Convers Manag* **209**, 112652 (2020).
18. Chum, H. *et al. Renewable Energy Sources and Climate Change Mitigation*. (Cambridge University Press, 2011). doi:10.1017/CBO9781139151153.
19. IEA Bioenergy. *Black Liquor Gasification*. (2013).
20. Gustafsson, M. & Svensson, N. Cleaner heavy transports – Environmental and economic analysis of liquefied natural gas and biomethane. *J Clean Prod* **278**, 123535 (2021).
21. Oosterkamp, W. J. Use of volatile solids from biomass for energy production. in *Recent Developments in Bioenergy Research* 131–145 (Elsevier, 2020). doi:10.1016/B978-0-12-819597-0.00006-4.
22. International Energy Agency. *Marine bunkers product demand - 2015-2024*. (2019).
23. IRENA. *Global Renewables Outlook: Energy transformation 2050*. (2020).
24. Moro, A. & Lonza, L. Electricity carbon intensity in European Member States: Impacts on GHG emissions of electric vehicles. *Transp Res D Transp Environ* **64**, 5–14 (2018).
25. European Parliament. *Greenhouse gas emissions from shipping: waiting for concrete progress at IMO level committee EN* . (2020).
26. UNEP/SETAC. *Global Guidance for Life Cycle Impact Assessment Indicators*. (2016).
27. International Energy Agency. *Net Zero by 2050*. (2021).
28. van Vuuren, D. P. *et al.* Energy, land-use and greenhouse gas emissions trajectories under a green growth paradigm. *Global Environmental Change* **42**, 237–250 (2017).
29. Laursen, R. Alternative Engine Development. (2021).

30. MAN Energy Solutions. The case for two-stroke ammonia engines. (2021).
31. Sørensen, T. A. & Laursen, R. *Total Cost of Ownership (TCO) - Sustainable Marine Fuels*. (2021).
